# A phase I open-label study of the safety and efficacy of apatinib (rivoceranib) administered to patients with advanced malignancies to improve sensitivity to pembrolizumab in the second- or later-line setting (APPEASE)

**DOI:** 10.1186/s13104-023-06283-5

**Published:** 2023-02-16

**Authors:** Matthew Gumbleton, Stephanie Allan, Hannah Conway, Kenneth Boucher, James Marvin, Josiah Hawks, William Burnett, Matthew Van Brocklin, Jonathan Whisenant, Glynn Gilcrease, Sumati Gupta

**Affiliations:** 1 Huntsman Cancer Institute, University of Utah, 2000 Circle of Hope Drive, Salt Lake City, UT USA; 2 Division of Oncology, Department of Internal Medicine, University of Utah School of Medicine, Salt Lake City, UT USA; 3 Division of Epidemiology and Huntsman Cancer Institute, Department of Internal Medicine, University of Utah School of Medicine, Salt Lake City, UT USA; 4 Flow Cytometry Core Facility, Health Science Center, University of Utah, Salt Lake City, UT USA; 5 Department of Oncological Sciences, University of Utah School of Medicine, Salt Lake City, UT USA; 6 Division of Oncology, Department of Surgery, University of Utah School of Medicine, Salt Lake City, UT USA; 7 Department of Veterans Affairs Medical Center, Salt Lake City, UT USA

**Keywords:** APPEASE, Apatinib, Rivoceranib, Pembrolizumab, Immunotherapy, VEGF inhibition

## Abstract

**Objective:**

APPEASE is a phase I study to assess the safety, dosing, and efficacy of rivoceranib (a selective, small-molecule inhibitor of VEGFR2) in combination with pembrolizumab. We aimed to treat patients with metastatic malignancies who have progressed through at least first-line therapy, with pembrolizumab 200 mg every 3 weeks, as well as escalating doses of rivoceranib until disease progression or unacceptable toxicity.

**Results:**

Five patients were enrolled on the starting dose of rivoceranib 300 mg once daily. There were no dose-limiting toxicities observed in combination with pembrolizumab. The dose of rivoceranib was not escalated due to study closure. We note a treatment related grade 3 adverse event (AE) rate of 40%, predominantly in urothelial cancer patients, with no deaths related to treatment related AEs. The disease control rate was 75% (3 of 4) and the median progression free survival (PFS) was 3.6 months. Tumor shrinkage was noted in patients who were previously progressing on pembrolizumab alone. Apatinib 300 mg is safe and demonstrates anti-tumor activity in advanced solid tumors in combination with pembrolizumab. Further dose escalation and efficacy need to be investigated in larger disease-specific patient populations.

*Trial registration number:* Clinical trial registration number: NCT03407976. Date of registration: January 17, 2018.

**Supplementary Information:**

The online version contains supplementary material available at 10.1186/s13104-023-06283-5.

## Introduction

Monoclonal antibodies targeting the programmed cell death protein-1 (PD-1)/programmed death-ligand 1 (PD-L1) have received Food and Drug Administration (FDA) approval for several malignancies and have established the important role for T-cell mediated host defense against malignancy [1]. While some patients with advanced metastatic upper gastrointestinal (GI) [2–4] or urothelial [5–7] malignancies achieve disease control with immune checkpoint blockade (ICB), the lack of durable response by most patients has prompted a search for adjunctive therapies to boost the effectiveness of ICB.

Rapid tumor growth results in a hypoxic tumor microenvironment. Tumors secrete vascular endothelial growth factor (VEGF), which binds its primary signaling receptor VEGF Receptor 2 (VEGFR2), promoting angiogenesis [8]. Neovascularization inhibits antitumor immunity through Fas ligand-mediated inhibition of effector CD8 cells and by sparing regulatory T cells (Tregs) [9]. VEGF directly inhibits the immune response by inhibiting the maturation of antigen-presenting cells (APCs), increased Treg recruitment, promoting macrophage repolarization to an M2 phenotype, and promoting T-cell exhaustion [10–12]. Inhibiting VEGF signaling may reverse this immunosuppressive phenotype. Indeed, several clinical trials have recently shown both improved median overall survival, as well as increased frequency of durable responses, amongst patients treated with anti-VEGF therapy in combination with ICB [13, 14].

Rivoceranib (apatinib) is a small-molecule selective inhibitor of VEGFR2 [15], and approved for use in China for metastatic / advanced gastric cancer. We report a phase I dose-escalation trial to evaluate the combination of pembrolizumab, an FDA-approved anti-PD-1 antibody, in combination with rivoceranib, in patients with previously treated metastatic malignancies adhering to the CONSORT guidelines.

## Main text

### Materials and methods

#### Study design and intervention

APPEASE was designed as a Phase I, single-site trial utilizing a standard 3 + 3 design, with the primary objective to assess the safety and maximum tolerated dose (MTD) of rivoceranib in combination with pembrolizumab in subjects with select advanced malignancies. The Phase I primary endpoints included adverse events (AEs), serious adverse events (SAEs), and the rate of dose-limiting toxicities (DLTs).

DLTs were defined as an AE causally related to rivoceranib and / or pembrolizumab during the 6 weeks of treatment (between the first day of the first cycle and the first day of the third cycle). Additionally, one of the following criteria had to be fulfilled: any grade 4 toxicity; grade 3 febrile neutropenia; grade 3 hematologic toxicity lasting > 7 days; uncontrollable hypertension defined as Stage 2 hypertension requiring a dose reduction; other grade 3 toxicities including nausea, vomiting, and diarrhea, continuing for more than 72 h despite optimal medical management; grade 2 toxicity warranting a preemptive dose reduction of rivoceranib in the opinion of the investigator; any toxicity of any grade warranting a dose reduction of rivoceranib; any toxicity of any grade warranting withholding or delaying a pembrolizumab infusion; immune-related toxicities which do not resolve to grade ≤ 1 within 14 days, including grade ≥ 2 pneumonitis, grade ≥ 2 enterocolitis, grade ≥ 2 nephritis, grade ≥ 2 hepatitis, grade ≥ 3 rash, grade ≥ 3 hypothyroidism, and any immune- related toxicity requiring systemic steroid treatment; Hy’s Law (3 × ULN elevation of transaminases and concomitant 2 × ULN elevation of bilirubin without alternative etiology); failure to receive at least 80% of the expected doses of rivoceranib due to toxicity.

The secondary objectives of the trial were to assess the efficacy of the combination. Objective response rate and progression-free survival were evaluated per Response Evaluation Criteria in Solid Tumors version 1.1 (RECIST 1.1) criteria.

Eligible patients were treated with 300 mg rivoceranib (LSK BioPartners) orally once daily, with a minimum of 16 h between doses, and 200 mg pembrolizumab IV infusion on the first day of each 21-day cycle. It was planned to increase the dose of rivoceranib by 100 mg, up to a maximum daily dose of 700 mg was reached, every three to six patients providing that at most one out of six patients treated experienced DLTs.

#### Inclusion criteria

Eligible patients aged 18 or over who qualified for pembrolizumab therapy based on FDA-approved indications were included: urothelial carcinoma having progressed during or following platinum-based chemotherapy; MSI-H or dMMR solid tumors; gastric or gastroesophageal junctio3n adenocarcinomas expressing PD-L1 (CPS ≥ 1) who had progressed on at least two prior lines of systemic chemotherapy, and, if appropriate, HER2 / neu-targeted therapy.

#### Study assessments

Primary objectives to assess the safety and tolerability of rivoceranib in combination with pembrolizumab were based on the rate of AEs, SAEs, and DLTs. All AEs and SAEs were graded with the National Cancer Institute Common Terminology Criteria for Adverse Events (v5.0).

Peripheral blood samples for correlative studies were collected before the beginning of the study drug treatment, after the completion of 2 cycles (21-day cycles), and at the time of progression or treatment discontinuation.

Disease assessments were performed every 9 weeks (± 1 week) with computed tomography scans of the chest, abdomen, and pelvis and treatment response was determined utilizing RECIST 1.1.

#### Flow cytometry

Peripheral blood mononuclear cells (PBMCs) were frozen at −80 °C and thawed immediately prior to analysis. Cells were washed and stained with Zombie UV fixable viability dye prior to surface marker staining (see Additional file 1: Table S1 for list of clones and manufacturers). Cells were analyzed using a 5 laser Cytek Aurora with SpectroFlow Software. Data analyzed with FlowJo V10.

#### Cytokine analysis

Serum samples were analyzed via ProcartaPlex Multiplex Immunoassay platinum panel 42-plex for humans according to the manufacturer’s instructions.

#### Statistical analysis

All statistical analyses were performed using the statistical software Prism (GraphPad Software, San Diego, CA), R version 4.0.2 (The Foundation for Statistical Computing, Vienna, Austria). or Stata release 13.1 (StataCorp, College Station, TX). Paired *t*-tests were used to compare changes in leukocyte populations over time. In all cases of multiple *t*-tests the two-stage step-up method of Benjamini, Krieger, and Yekutieli was used to correct for multiple comparisons.

## Results

### Patient characteristics

Five patients enrolled from 8 /4 /2018 to 7/8/2019 to dose Level 1 and the last follow-up was performed on 7 /2/2020. The baseline characteristics are described in Table 1. The study closed to accrual on 09 /11/2019 benefactor’s decision not to proceed with the study.

**Table 1:** Baseline demographics and characteristics

	Rivoceranib + Pembrolizumab (*n* = 5)
Age, median (range), in years	65 (52–72)
Male, *n* (%)	4 (80%)
Diagnosis	
Urothelial carcinoma of the bladder	2 (40%)
Gastric and gastroesophageal adenocarcinoma	3 (60%)
MSI– High	0 (0%)
Sites of metastasis	
Lung	2 (40%)
Lymph Node	2 (40%)
Liver	1 (20%)
Bone	1 (20%)
ECOG	
0	3 (60%)
1	2 (40%)
Former smoker	2 (40%)
History of hypertension	3 (60%)
Prior cancer therapy	
Surgery	3 (60%)
Radiation	4 (80%)
Chemotherapy	5 (100%)
Immunotherapy	3 (60%)
Pembrolizumab	2 (40%)
Targeted	3 (60%)

### Toxicity

Three patients were initially accrued to dose level 1 (rivoceranib 300 mg daily). These patients did not have any DLT. However, due to AEs of hypertension, fatigue and nephritis observed outside the DLT period requiring dose holds and dose reductions, the dose of rivoceranib was not escalated above 300 mg daily and 2 more patients were accrued to dose level 1.

There were no DLTs noted in any of the five patient treated on this phase 1 study. All study participants experienced adverse events related to study therapy as assessed by the treating investigator (Table 2); one patient had significant nausea prior to trial enrollment, which remained uncontrolled while on trial leading to withdrawal. Table 2 provides a summary of the toxicity profile observed in the study population; detailed summaries of adverse events attributed to study therapy are shown in Additional file 2: Table S2. Grade 3 AEs attributed to rivoceranib alone were hypertension, anemia, sepsis and increased INR, and predominated in patients with urothelial carcinoma.

**Table 2:** Toxicity profile and safety summary

Feature	Frequency
Number of cycles received, mean (range)	4 (2–6)
All–grade AEs, any cause, *n* (%)	5 (100%)
Treatment–related AE	5 (100%)
Immune–related AE	3 (60%)
Grade 3 AEs, any cause, *n* (%)	2 (40%)
Treatment–related grade 3 AEs	2 (40%)
Treatment–related AEs leading to withdrawal from treatment	1 (20%)
AEs leading to dose modification or interruption, *n* (%)	3 (60%)
AEs leading to death, *n* (%)	0 (0%)

### Efficacy

Four out of the five patients on the study had measurable disease as defined by RECIST 1.1 at the time of enrollment. The patient without measurable disease discontinued study therapy early due to poor tolerance. The patient did not have a disease assessment while on study, and therefore was excluded from all efficacy analyses except overall survival. On average, patients completed 4 cycles of therapy with a range from 2 to 6 cycles. However, at the time of study discontinuation, one subject transferred over to an expanded access program and received an additional 4 cycles of the combination, receiving a total of 6 cycles of study therapy.

**Fig. 1:**
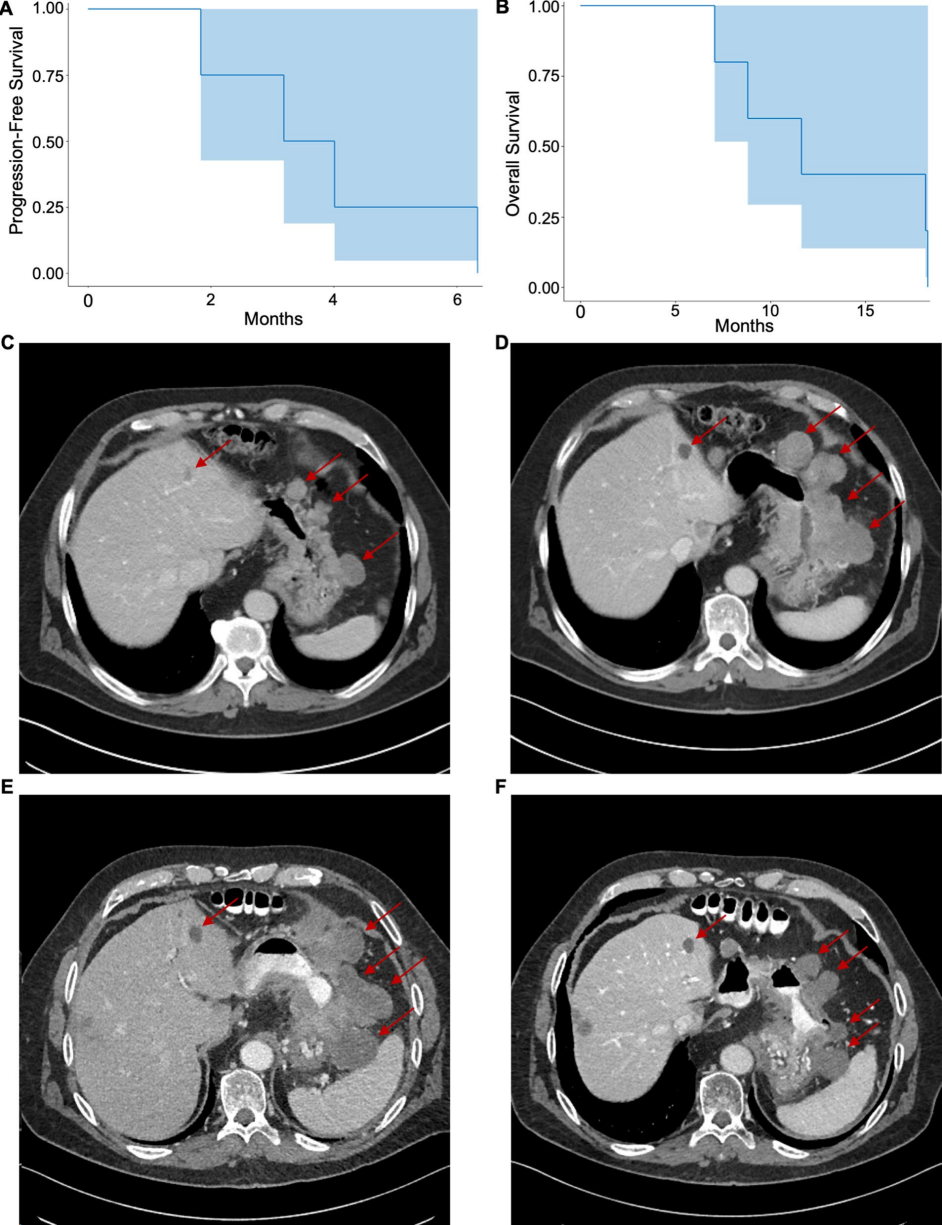
Response to treatment with pembrolizumab + rivoceranib. Kaplan-Meier Curves demonstrating **A** Progression Free Survival as well as **B** Overall survival. The shaded area represents the pointwise 95% confidence interval. Representative CT images from a patient with metastatic Her2-overexpressed gastric adenocarcinoma **C** 12 / 27 / 2018 CT abdomen demonstrated initial progression following mFOLFOX6 while on Herceptin maintenance prior to starting second line Pembrolizumab. **D** Restaging imaging 4 / 9 / 2019 following cycle 4 Pembrolizumab demonstrating continued progression. **E **Restaging CT abdomen 7/ 1 /2019, one week prior to cycle one Pembrolizumab + Rivoceranib. **F** Restaging CT abdomen 9/6 /2019 demonstrating reduction in size of multiple sites of metastatic disease while on trial

Three of the four patients with measurable disease achieved stable disease with disease shrinkage on scans while on study therapy, two of these had previously progressed on pembrolizumab. One patient had disease progression without any appreciable response. As shown in Fig. 1A, B, the median progression-free survival (PFS) was 3.6 months, and the median overall survival (OS) was 11.6 months. Interestingly, prior to enrolling in APPEASE, one patient with metastatic gastric adenocarcinoma had received 4 cycles of second-line pembrolizumab monotherapy without any evidence of response (Fig. 1C–E). Following the addition of rivoceranib a reduction in the size of multiple metastatic lesions was observed (Fig. 1F).

### Immunomodulatory effects

We analyzed the treatment effect on the peripheral blood lymphoid and myeloid compartments, as well as serum cytokine levels by evaluating these at screening, at the start of cycle 3, and at the end of treatment visit. Luo et al. recently described a small increase in Treg frequency one month after patients started on rivoceranib 500 mg daily [16]. Preclinical data has not demonstrated any difference in Treg frequency with rivoceranib treatment [17] Consistent with this, we did not find any change in Treg homeostasis after starting treatment, and these populations were also unchanged after discontinuing therapy (Additional file 3: Fig. S1). Other major lymphoid and myeloid populations also did not have altered homeostasis while on rivoceranib (Additional file 3: Fig. S1).

We analyzed peripheral blood lymphocyte expression of a panel of immune checkpoints, as well as costimulatory receptors and markers of activation (Additional file 4: Fig. S2). Given the small number of patients and the large panel of surface markers, most changes were not statistically significant after correcting for multiple observations. Interestingly, we found that the patient with metastatic gastric adenocarcinoma (scans shown in Fig. 1C–F) had a large decrease in frequency CD4^+^ T-cell, CD8^+^ T-cell, and NK cell with expression of multiple immune checkpoints including LAG3 and TIM3 (Additional file 3: Fig. S1D, F), as well as decreased CD95 expression amongst both CD4^+^ and CD8^+^ T-cell subsets after starting on trial therapy ((Additional file 4: Fig. 2SH). After discontinuing therapy, there was a significant increase in the frequency of CD8^+^GITR^+^ peripheral blood T-cells (Additional file 4: Fig. S2).

We did not find significant changes in serum cytokine levels while on rivoceranib and pembrolizumab (Additional file 5: Fig. S3).

## Discussion

We designed this single-arm Phase I trial to understand the safety and tolerability of treatment with rivoceranib in combination with the FDA-approved anti-PD1 antibody pembrolizumab. Rivoceranib increases antitumor immunity when combined with the non-FDA approved anti-PD1 antibody camrelizumab in several contexts [18–23].

We did not observe any DLTs in the five patients treated with rivoceranib 300 mg once a day and pembrolizumab 200 mg every 3 weeks, although several patients did experience treatment-related AEs. Hence, we expanded the enrollment of patients at dose level 1 (300 mg). Despite doses of up to 850 mg rivoceranib daily, as a single agent, in several previous trials focused on gastric cancer, patients with metastatic urothelial carcinoma, had difficulty tolerating 300 mg rivoceranib daily in combination with pembrolizumab beyond the initial DLT period in this trial. All toxicities were manageable with no treatment-related deaths.

In this heavily-pretreated population of patients, 3 of 4 patients (those with measurable disease burden) received clinical benefit and had stable disease with disease shrinkage while on trial, including two patients who had previously progressed on pembrolizumab, one of which had a significant reduction in metastatic burden as shown in Fig. 1. Interestingly, this patient had a very large reduction in NK cell populations that express multiple immune checkpoints, as well as a large reduction in expression of multiple immune checkpoints amongst his CD8 + effector T-cells. To our knowledge, this is also the broadest analysis of the effect on the peripheral blood lymphocyte and myeloid compartments in response to inhibiting these two signaling receptors to date.

In conclusion, amongst a heavily pretreated population of patients with metastatic urothelial, gastric and gastroesophageal adenocarcinoma, rivoceranib 300 mg daily combined with pembrolizumab can be used safely. Moreover, the majority of patients received clinical benefit from treatment, and none experienced any DLTs.

## Limitations

In the heterogeneous population of advanced /metastatic gastroesophageal, gastric and urothelial cancers differential tolerance to study medications was noted. In this small study with five patients, we did not escalate to higher doses due to early study closure. This treatment strategy deserves further dosing and efficacy investigation in larger disease-specific patient populations.

## Supplementary Information

**Additional file 1: Table S1. **Antibodies used for flow cytometry analysis. 

**Additional file 2: Table S2. **Study therapy related adverse events. 

**Additional file 3: Fig S1.  **Peripheral blood lymphoid and myeloid homeostasis was stable after starting rivoceranib + pembrolizumab.  

**Additional file 4: Fig S2. **Immune checkpoint expression, costimulatory molecule expression, and activation marker expression was largely unchanged while on apatinib and pembrolizumab. 

**Additional file 5: Fig S3. **Peripherial blood cytokine levels were unchanged while on apatinib and pembrolizumab. 

## Data Availability

All data are available upon request.

## Abbreviations

GI: Gastrointestinal
PD-1: Programmed cell death protein-1
PD-L1: Programmed death-ligand 1
TMB: Tumor mutational burden
ICB: Immune checkpoint blockade
VEGF: Vascular endothelial growth factor
VEGFR2: Vascular endothelial growth factor receptor 2
Treg: Regulatory T cells
APCs: Antigen-presenting cells
FDA: Food and drug administration
MTD: Maximum tolerated dose
AEs: Adverse events
SAEs: Serious adverse events
DLTs: Dose-limiting toxicities
ULN: Upper limit of normal
RECIST 1.1: Response Evaluation Criteria in Solid Tumors version 1.1
MSI-H: Microsatellite instability-high
dMMR: Deficient DNA mismatch repair
HER2: Human Epidermal growth factor Receptor 2
PBMCs: Peripheral blood mononuclear cells
