# Supplementary material for: A phase I open-label study of the safety and efficacy of apatinib (rivoceranib) administered to patients with advanced malignancies to improve sensitivity to pembrolizumab in the second- or later-line setting (APPEASE)
Source: BMC Res Notes. 2023 Feb 16;16:16. doi: 10.1186/s13104-023-06283-5 (PMC9936706; doi:10.1186/s13104-023-06283-5)
Supplement: Supplementary file 1 — Additional file 1: Table S1. Antibodies used for flow cytometry analysis. [file 13104_2023_6283_MOESM1_ESM.docx]

**Table S1. Antibodies used for flow cytometry analysis.**

| **Antigen** | **Fluorophore** | **Clone** | **Vendor** | |  |
| --- | --- | --- | --- | --- | --- |
| CD69 | BV750 | FN50 | BD | |  |
| HLA‐DR | APC‐R700 | G46–6 | BD | |  |
| CD183 (CXCR3) | BV650 | G025H7 | BL | |  |
| CD186 (CXCR6) | BUV661 | 13B 1E5 | BD | |  |
| CD19 | BUV615 | H1B19 | BD | |  |
| CD244 (2B4) | PECY7 | C1.7 | BL | |  |
| LAG3 | PE‐dazzle 594 | 3DS223H | BL | |  |
| CD279 (PD‐1) | BV785 | EH12.2H7 | BL | |  |
| CD366 (TIM‐3) | BB515 | 7D3 | BD | |  |
| CD134 (OX40) | BV711 | L106 | BL | |  |
| CD137 (4–1BB) | BV605* | 4B4–1 | BL | |  |
| CD278 (ICOS) | BV421 | DX29 | BD | |  |
| CD357 (GITR) | APC‐Fire 750 | 108–17 | BL | |  |
| CD56 | Percp | NCAM | BL | |  |
| CD127 (IL‐7Rα) | PE‐Cy5 | A019D5 | BL | |  |
| CD197 (CCR7) | BUV395 | 150503 | BD | |  |
| CD25 | BUV563 | 2A3 | BD | |  |
| CD28 | BUV805 | L293 | BD | |  |
| CD45RO | APC | UCHL1 | BD | |  |
| CD95 | BV480 | DX2 | BD | |  |
| CD27 | PE | O323 | BL | |  |
| CD3 | BUV496 | UCHT1 | BD | |  |
| CD4 | BUV737 | SK3 | BD | |  |
| CD8 | BV570 | RPA‐T8 | BL | |  |
| Amine‐Reactive | LIVE/DEAD Blue | N/A | TF | |  |
| **Antigen** | **Fluorophore** | **Clone** | | **Vendor** | |
| CD56 | PerCP | NCAM16.2 | | BL | |
| CD3 | A647 | UCHT1 | | BL | |
| CD19 | BUV615 | SJ25‐C1 | | BD | |
| CD14 | BV711 | MφP9 | | BL | |
| HLADR | APC‐Fire 750 | G46‐6 | | BL | |
| CD11c | AF700 | B‐ly6 | | BL | |
| CD123 | BV785 | 7G3 | | BL | |
| CD33 | BV 421 | WM53 | | BL | |
| CD16 | BUV496 | 3G8 | | BD | |
| CD15 | BV510 | W6D3 | | BL | |
| CD64 | BV605 | 10.1 | | BL | |
| CD11b | BV750 | ICRF44 | | BL | |
| Dead cells | LIVE/DEAD Blue |  | | TF | |
| CD45 | BUV805 | HI30 | | BD | |
