# Supplementary material for: A phase I open-label study of the safety and efficacy of apatinib (rivoceranib) administered to patients with advanced malignancies to improve sensitivity to pembrolizumab in the second- or later-line setting (APPEASE)
Source: BMC Res Notes. 2023 Feb 16;16:16. doi: 10.1186/s13104-023-06283-5 (PMC9936706; doi:10.1186/s13104-023-06283-5)
Supplement: Supplementary file 2 — Additional file 2: Table S2. Study therapy related adverse events. [file 13104_2023_6283_MOESM2_ESM.docx]

**Table S2: Study therapy related adverse events**

| **Adverse Event** | **Immune-Related** | **Any Grade**  n (%) | **Grade 1/2**  n (%) | **Grade 3**  n (%) | **Attributed to Rivoceranib** | **Attributed to Pembro** |
| --- | --- | --- | --- | --- | --- | --- |
| **Blood and lymphatic system disorders** | | | | | | |
| Anemia | No | 1 (20%) | 1 (20%) | 1 (20%)*^ϯ^ | Yes | No |
| **Endocrine disorders** | | | | | | |
| Hypothyroidism | No | 1 (20%) | 1 (20%) | 0 | Yes | Yes |
| **Gastrointestinal disorders** | | | | | | |
| Diarrhea | Yes | 2 (40%) | 2 (40%) | 0 | Yes | Yes |
| Dysphagia | No | 1 (20%) | 1 (20%) | 0 | Yes | No |
| Nausea | No | 1 (20%) | 1 (20%)^ϯ^ | 0 | Yes | No |
| **General disorders and administration site conditions** | | | | | | |
| Fever | Yes | 1 (20%) | 1 (20%)^ϯ^ | 0 | No | Yes |
| Fatigue | No | 1 (20%) | 1 (20%) | 0 | Yes | Yes |
| **Infections and infestations** | | | | | | |
| Sepsis | No | 1 (20%) | 0 | 1 (20%) | Yes | No |
| **Injury, poisoning, and procedural complications** | | | | | | |
| Urostomy site bleeding | No | 1 (20%) | 0 | 0 | Yes | No |
| **Investigations** | | | | | | |
| INR increased | No | 1 (20%) | 0 | 1 (20%) | Yes | No |
| **Musculoskeletal and connective tissue disorders** | | | | | | |
| Pain in extremity | No | 1 (20%) | 1 (20%) | 0 | Yes | No |
| **Nervous system disorders** | | | | | | |
| Headache | No | 1 (20%) | 1 (20%) | 0 | Yes | No |
| **Renal and urinary disorders** | | | | | | |
| Acute kidney injury | Yes | 1 (20%) | 1 (20%)^ϯ^ | 0 | No | Yes |
| Proteinuria | No | 1 (20%) | 1 (20%)^ϯ^ | 0 | No | Yes |
| Nephritis | Yes | 1 (20%) | 1 (20%) | 1 (20%)* | No | Yes |
| **Respiratory, thoracic and mediastinal disorders** | | | | | | |
| Epistaxis | No | 1 (20%) | 1 (20%) | 0 | Yes | No |
| Hoarseness | No | 1 (20%) | 1 (20%) | 0 | Yes | No |
| **Skin and subcutaneous tissue disorders** | | | | | | |
| Pruritus | Yes | 2 (40%) | 2 (40%) | 0 | No | Yes |
| Rash maculopapular | Yes | 1 (20%) | 1 (20%) | 0 | No | Yes |
| Palmar-plantar erythrodysesthesia syndrome | No | 1 (20%) | 1 (20%) | 0 | Yes | No |
| **Vascular disorders** | | | | | | |
| Hypertension | No | 3 (60%) | 3 (60%) | 1 (20%)* | Yes | No |
| * represents a single worsening patient  ^ϯ^ lead to study therapy interruption, modification, or discontinuation in a single patient | | | | |  |  |
