# Supplementary material for: A phase I open-label study of the safety and efficacy of apatinib (rivoceranib) administered to patients with advanced malignancies to improve sensitivity to pembrolizumab in the second- or later-line setting (APPEASE)
Source: BMC Res Notes. 2023 Feb 16;16:16. doi: 10.1186/s13104-023-06283-5 (PMC9936706; doi:10.1186/s13104-023-06283-5)
Supplement: Supplementary file 3 — Additional file 3: Fig S1. Peripheral blood lymphoid and myeloid homeostasis was stable after starting rivoceranib + pembrolizumab. [file 13104_2023_6283_MOESM3_ESM.docx]

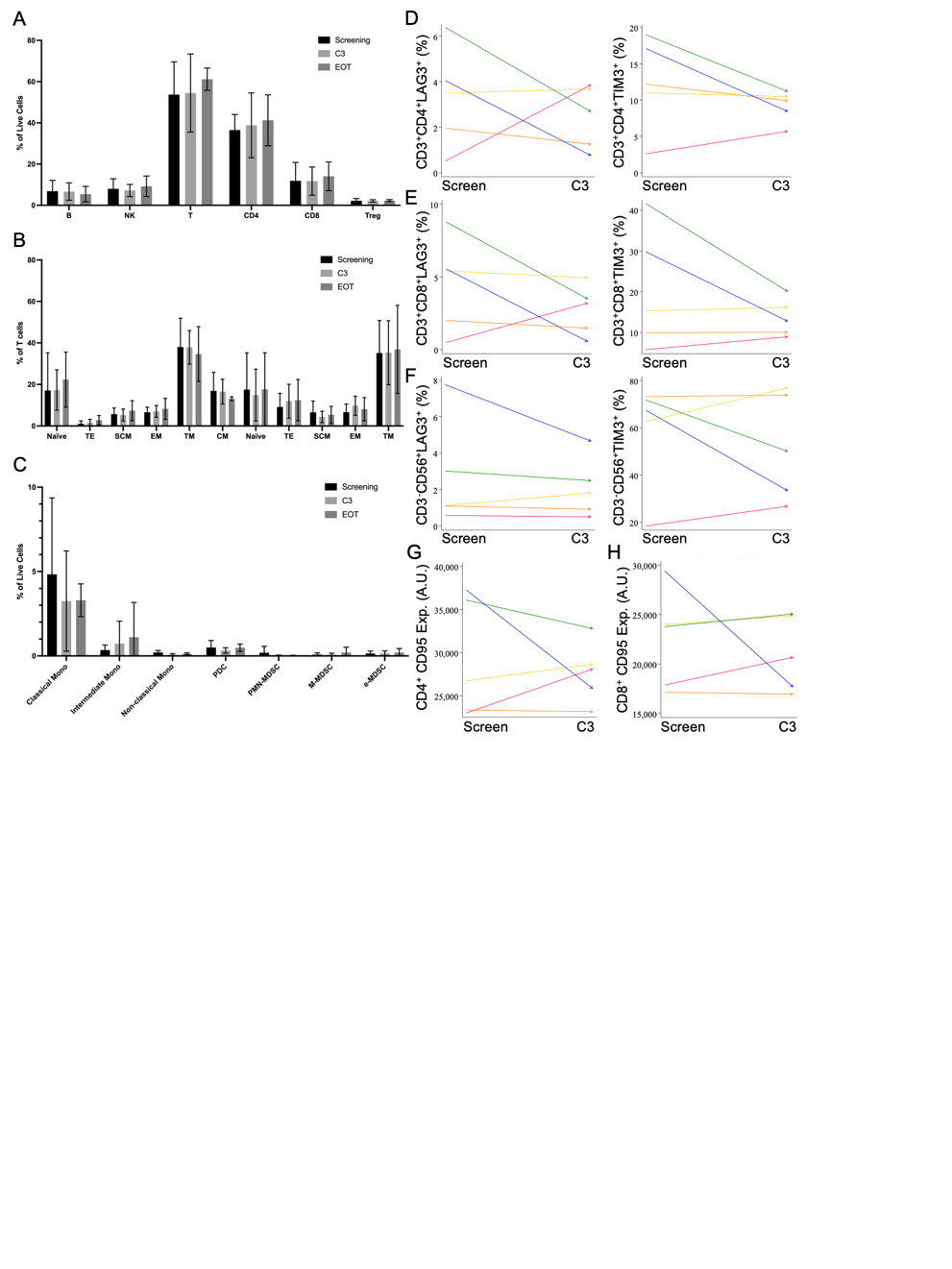


**Fig S1 Peripheral blood lymphoid and myeloid homeostasis was stable after starting rivoceranib + pembrolizumab**. Peripheral blood samples were obtained prior to starting therapy (Dark bars), at the start of cycle 3 (lightest bars), or at end of treatment visit (grey bars), and analyzed by flow cytometry to determine (A) T-cell, B-cell and NK-cell homeostasis, as well as (B) frequency of T cell subsets, and (C) myeloid homeostasis. The patient (blue line) with metastatic gastric adenocarcinoma demonstrated decreased frequency of (D) CD4^+^ T-cell, (E) CD8^+^ T-cell, (F) and NK cell expressing the immune checkpoints TIM3 and LAG3, as well as decreased expression of CD95 (Fas) amongst (G) CD4^+^ T-cells as well as (H) CD8^+^ T-cells
