# Supplementary material for: A phase I open-label study of the safety and efficacy of apatinib (rivoceranib) administered to patients with advanced malignancies to improve sensitivity to pembrolizumab in the second- or later-line setting (APPEASE)
Source: BMC Res Notes. 2023 Feb 16;16:16. doi: 10.1186/s13104-023-06283-5 (PMC9936706; doi:10.1186/s13104-023-06283-5)
Supplement: Supplementary file 4 — Additional file 4: Fig S2. Immune checkpoint expression, costimulatory molecule expression, and activation marker expression was largely unchanged while on apatinib and pembrolizumab. [file 13104_2023_6283_MOESM4_ESM.docx]

**
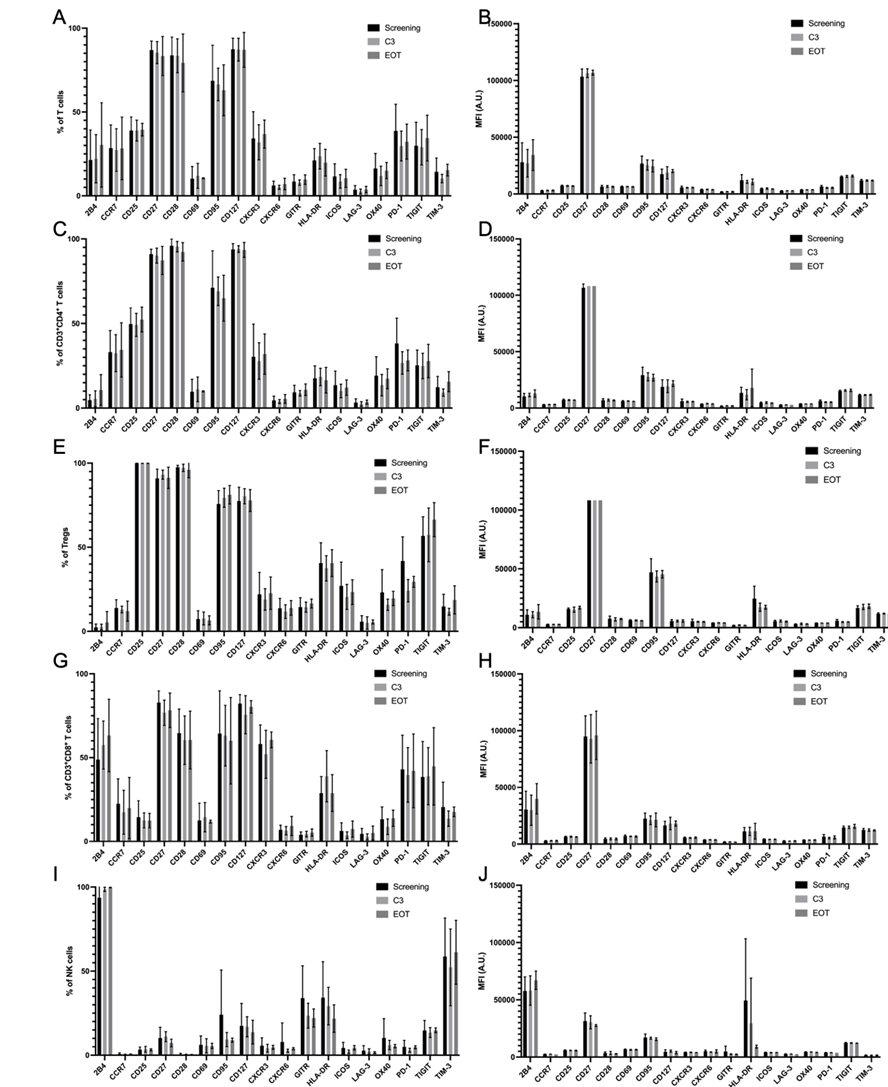
**

**Fig S2 Immune checkpoint expression, costimulatory molecule expression, and activation marker expression was largely unchanged while on apatinib and pembrolizumab.** Peripheral blood samples from prior to starting therapy (Dark bars), at the start of cycle 3 (lightest bars), or at end of treatment visit (grey bars), and analyzed by flow cytometry for expression of immune checkpoints, costimulatory molecules, and activation markers to determine (A, C, E, G, I) frequency, and expression level (B, D, F, H, J) amongst (A, B) T-cells (C, D) CD4^+^ T-cells, (E, F) regulatory T-cells, (G, H) CD8^+^ T-cells, and (I, J) NK-cells
