# Supplementary material for: A phase I open-label study of the safety and efficacy of apatinib (rivoceranib) administered to patients with advanced malignancies to improve sensitivity to pembrolizumab in the second- or later-line setting (APPEASE)
Source: BMC Res Notes. 2023 Feb 16;16:16. doi: 10.1186/s13104-023-06283-5 (PMC9936706; doi:10.1186/s13104-023-06283-5)
Supplement: Supplementary file 5 — Additional file 5: Fig S3. Peripherial blood cytokine levels were unchanged while on apatinib and pembrolizumab. [file 13104_2023_6283_MOESM5_ESM.docx]

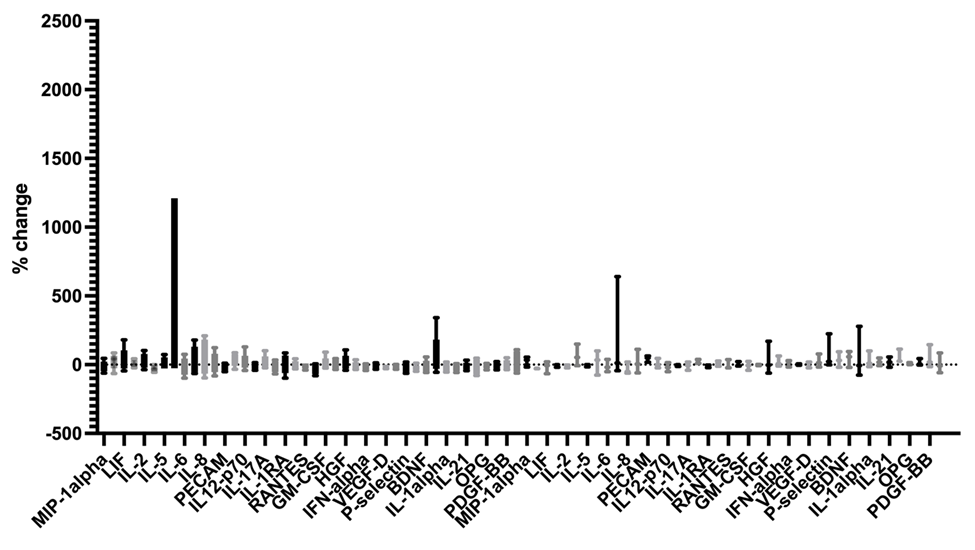


**Fig S3 Peripherial blood cytokine levels were unchanged while on apatinib and pembrolizumab.** Peripheral blood samples were collected prior to starting therapy (Dark bars), at the start of cycle 3 (lightest bars), or at end of treatment visit (grey bars), and analyzed by the ProcartaPlex Multiplex Immunoassay platinum panel 42-plex
